# External Venous Drainage (EVD) System: A Standardized Bedside Technique for Venous Congestion in Free Flaps

**DOI:** 10.1002/ccr3.73094

**Published:** 2026-07-16

**Authors:** Marco Marcasciano, Jacopo Nanni, Chad Chang, Guido Firmani, Yasser Farid, Iván Enrique Rodríguez‐Mantilla, Alex Sorkin, Hung‐Chi Chen

**Affiliations:** ^1^ Division of Plastic and Reconstructive Surgery, Department of Surgery China Medical University Hospital Taichung Taiwan; ^2^ Plastic and Reconstructive Surgery Unit, Department of Experimental and Clinical Medicine “Magna Graecia” University of Catanzaro Catanzaro Italy; ^3^ Department of Plastic and Reconstructive Surgery Royal Victoria Infirmary Newcastle Upon Tyne UK; ^4^ Department of Plastic and Reconstructive Surgery, Sant'Andrea Hospital, NESMOS (Neurosciences, Mental Health and Sensory Organs) Department Sapienza University of Rome Rome Italy; ^5^ Department of Plastic and Reconstructive Surgery Brugmann Hospital Brussels Brussels Belgium; ^6^ Department of Plastic and Reconstructive Surgery, Hospital de San José Fundación Universitaria de Ciencias de la Salud Bogotá D.C Colombia; ^7^ Department of Plastic Surgery Shamir Medical Center Tel Aviv Israel

**Keywords:** external venous drainage, flap salvage, microsurgical reconstruction, venous congestion

## Abstract

In cases of venous congestion where re‐exploration is not feasible, the external venous drainage (EVD) system provides a simple and standardized approach to maintain venous outflow, supporting flap viability through controlled dermal bleeding and local anticoagulation.

## 1 Introduction

Venous congestion remains a major cause of failure in microsurgical reconstruction and replantation, occurring in up to 5% of cases despite technical advancements [1, 2, 3]. Overall free flap failure rates are reported between 2% and 5%, with venous insufficiency representing one of the leading causes of flap compromise [4]. The window for salvage is narrow, as irreversible microvascular damage may occur within hours. When surgical revision is not feasible or additional venous outflow cannot be established, non‐surgical measures become critical. Hirudotherapy is widely used and can be effective in relieving venous congestion through mechanical blood removal and the anticoagulant properties of leech saliva. However, it is associated with variable tolerance, limited control of targeted areas and risk of *Aeromonas hydrophila* infection [5, 6, 7]. Ethical and ecological concerns further limit its availability in some regions [8]. In addition, imprecise localization and the need for repeated application may reduce its practicality in selected clinical scenarios. Beyond early‐onset congestion, re‐exploration may be infeasible or predictably ineffective due to the absence of usable venous drainage in the flap or recipient site [9]. Similarly, in large or extended flaps where distal venous stasis occurs and supercharging is not technically possible, a controlled bedside method of venous offloading is essential. In these contexts, alternative strategies capable of providing continuous and localized venous drainage are needed. This paper describes the External Venous Drainage (EVD) system and a practical algorithm for its application. The aim is to present a standardized and reproducible technical approach based on established principles of dermal bleeding and local anticoagulation.

## 2 Case Investigations and Treatment

**FIGURE 1:**
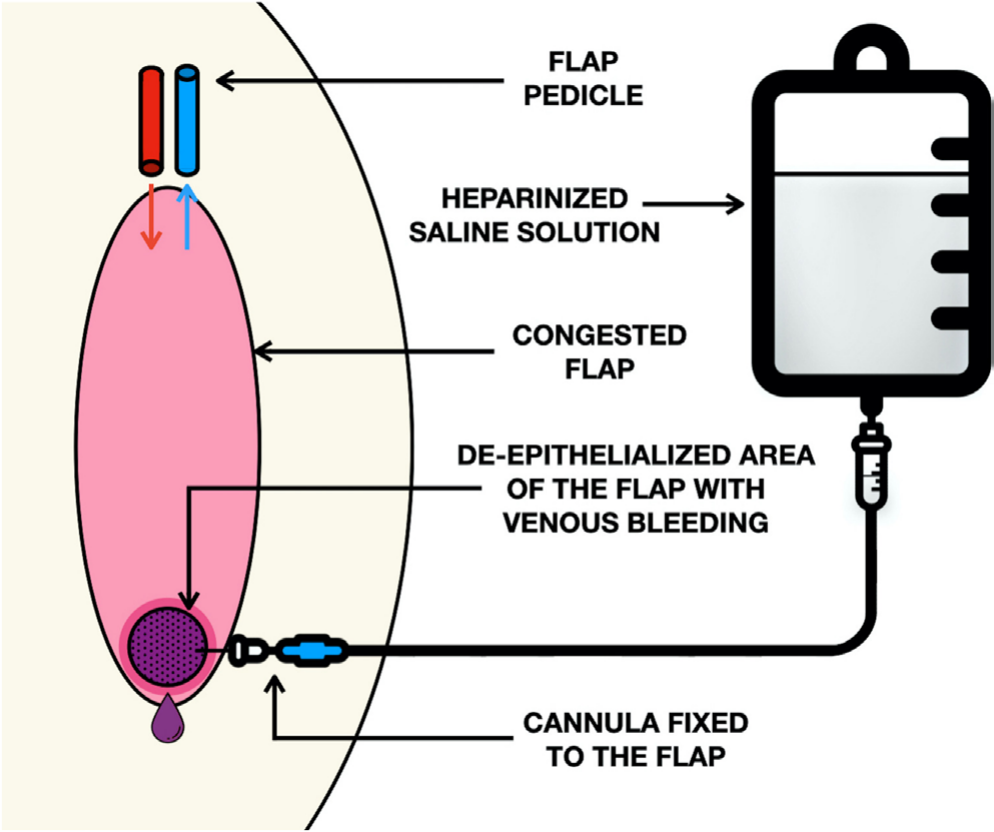
Schematic representation of the EVD system and its components.

The EVD consists of a disposable winged intravenous cannula connected to a continuous infusion of heparinized saline (5000 IU heparin in 500 mL). A small 2 × 1.5 cm de‐epithelialized area is created in the most dependent or congested region of the flap. (Figure 1) This limited size allows sufficient dermal bleeding for venous decompression while minimizing discomfort, fluid loss, and scarring, thereby optimizing the aesthetic outcome. Enoxaparin (low‐molecular‐weight heparin, LMWH) is injected once daily into the dermis of the de‐epithelialized area at a dose of 20–60 mg, individualized according to flap size, patient weight, and thrombotic risk [10]. This approach uses the same LMWH employed for systemic prophylaxis while exploiting the flap as a localized site of administration, providing an additional empiric local anticoagulant effect. The irrigation rate is set at 62.5 mL/h (approximately 1 mL/min), maintaining a continuously moist surface while avoiding pooling or tissue maceration. At this rate, a 500 mL solution bag lasts approximately 8 h. Flow is regulated using an infusion set with a flow controller and fine adjustment via roller clamp. The cannula is positioned tangentially to the flap to ensure even saline dispersion, while adjacent sterile gauze functions as a wick to prevent localized pooling. The device is typically maintained for 7–10 days, corresponding to the period required for neovascularization and resolution of venous congestion [11]. Continuous irrigation helps prevent clot formation, and minor fibrin deposits are gently removed during dressing changes (Video 1). Blood loss is monitored through daily hemoglobin measurements integrated into routine fluid balance assessment and transfusion is reserved for patients meeting institutional thresholds. Regular nursing surveillance ensures dressing integrity and prevents maceration.

**VIDEO 1:** Clinical application of the EVD system in a congested flap. Video content can be viewed at https://onlinelibrary.wiley.com/doi/10.1002/ccr3.73094.

## 3 Discussion

Venous congestion from thrombosis or impaired outflow poses an immediate threat to flap viability [1]. While surgical re‐exploration remains the gold standard, anatomical, logistical, or systemic factors may preclude timely intervention, leaving bedside medical management as the only salvage option [2]. Hirudotherapy alleviates congestion by exploiting the leech's natural anticoagulant properties and mechanical blood removal [12]. However, leech migration limits precise targeting, particularly in large or extended flaps dependent on a small vein. The EVD represents an alternative to leech therapy and a temporizing or adjunctive strategy, maintaining controlled venous offloading through sustained dermal bleeding until spontaneous recovery or surgical revision becomes possible. The combination of de‐epithelialization and topical heparin is well established in microsurgical practice, traditionally performed using heparin‐soaked pledgets replaced frequently [13, 14]. The EVD standardizes and refines this principle by providing continuous irrigation and consistent anticoagulant exposure without repeated manual intervention. The small de‐epithelialized window ensures effective drainage while limiting aesthetic morbidity. By avoiding leech handling [15], the EVD mitigates infection risk, improves patient comfort, and eliminates pain, anxiety, and psychological distress, thereby enhancing compliance. Several mechanical and chemical alternatives have been described [16, 17, 18], including venous cannulation needles [19], continuous suction [20, 21], pulsatile suction with mechanical agitation [22, 23, 24], and subcutaneous anticoagulation [25] (Table 1). These methods are often complex, cumbersome, and lack sustained efficacy. Our device represents an evolution of “concept 3” described by Conforti et al. [25] requiring no specialized equipment, prototypes or hand‐crafted instruments, making it practical and ready deployable. Unlike other systems that demand constant supervision and disposal, the EVD is compact, portable, and easily integrable into routine clinical workflows. Within our algorithm, EVD may be used prophylactically in cases of suspected venous impairment and in early clinically evident congestion when immediate re‐exploration is not feasible. Local LMWH administration complements surface irrigation by providing anticoagulation within the dermis and subdermis, reducing the risk of thrombus propagation.

**TABLE 1:** Comparison of mechanism of action, advantages and disadvantages of established systems for managing venous congestion.

	Mechanism of action	Advantages	Disadvantages
Medical leech therapy	Natural anticoagulation Blood loss by feeding	Effective Demonstrated results in humans	Expensive (10$/piece) Infective risk Effective only well‐vascularized flap parts Requires change after each feeding Psychologically distressing
Chemical alternatives	Anticoagulation	Less invasive	Inconsistent effectiveness
Mechanical alternatives	Mechanical suction	Less distressing	Cumbersome Expensive
External venous drainage system	De‐epithelization and local anticoagulation	Cheap Easily replicable	Not suitable for buried flaps Requires frequent dressing changes

Empirical use of LMWH in flap congestion is supported by existing literature, and though standardized dosing is lacking, the physiologic rationale aligns with enhancing dermal venous outflow and mitigating intradermal thrombosis [11, 26, 27]. In this context, dosing should be individualized based on patient characteristics and flap size, with careful clinical monitoring. The selected irrigation rate of 62.5 mL/h aligns with continuous irrigation systems used in other surgical contexts (typically 50–100 mL/h) [28, 29]. This rate maintains adequate surface hydration for sustained oozing while minimizing maceration. Although minor clotting can occur, gentle debridement during dressing changes restores function. The 2 × 1.5 cm de‐epithelialized window reflects a balance between effective bleeding and minimal scarring. Larger wounds do not improve efficacy and increase bleeding risk and postoperative scarring. Clinically, the EVD has demonstrated utility in cases when immediate surgical revision was not possible or when venous re‐anastomosis was impractical due to lack of suitable recipient veins. It also has value in extended flaps with distal congestion where supercharging is unfeasible, and in non‐microsurgical settings such as nipple–areola complex congestion after breast surgery or pedicled flaps [30]. The algorithm emphasizes early reassessment, and lack of clinical improvement should prompt consideration of surgical re‐exploration, particularly in large or composite flaps. In delayed congestion (> 5–6 days postoperatively), the decision to re‐explore must balance neovascularization against thrombosis extension risk. This report has several limitations. It does not include systematically collected quantitative clinical outcome data or comparative analysis with established techniques such as hirudotherapy. As such, conclusions regarding efficacy remain preliminary and should be interpreted with caution. Additionally, the safety profile of local LMWH administration, although supported by pharmacologic rationale, requires further validation in larger clinical studies. Despite these limitations and although not conceptually novel, the EVD system provides a standardized and reproducible technical approach that may facilitate controlled venous offloading in selected clinical scenarios. Its simplicity and accessibility may support broader adoption, particularly in resource‐limited settings or when conventional options are contraindicated or unavailable.

## 4 Conclusion

The external venous drainage (EVD) system represents a simple, standardized, and readily deployable technique for the management of venous congestion in both microsurgical and non‐microsurgical settings when surgical re‐exploration is not feasible. By integrating de‐epithelialization, continuous topical anticoagulation, and local LMWH administration, it provides a controlled method for venous offloading based on established physiologic principles. While the technique appears promising, further prospective studies are required to evaluate its clinical efficacy, define optimal protocols, and assess safety outcomes in a systematic manner.

## Author Contributions

**Marco Marcasciano:** conceptualization, funding acquisition. **Jacopo Nanni:** conceptualization, data curation. **Chad Chang:** project administration, resources. **Guido Firmani:** conceptualization, resources. **Yasser Farid:** conceptualization. **Iván Enrique Rodríguez‐Mantilla:** conceptualization, resources. **Alex Sorkin:** conceptualization, resources. **Hung‐Chi Chen:** conceptualization.

## Funding

The authors have nothing to report.

## Ethics Statement

The study was conducted in accordance with the Declaration of Helsinki.

## Consent

Written informed consent for participation and publication was obtained from all patients.

## Conflicts of Interest

The authors declare no conflicts of interest.

## Data Availability

Data sharing not applicable to this article as no datasets were generated or analysed during the current study.
